# Estrogen receptors genes polymorphisms and age at menarche in idiopathic scoliosis

**DOI:** 10.1186/1471-2474-15-383

**Published:** 2014-11-19

**Authors:** Piotr Janusz, Malgorzata Kotwicka, Miroslaw Andrusiewicz, Dariusz Czaprowski, Jaroslaw Czubak, Tomasz Kotwicki

**Affiliations:** Department of Pediatric Orthopedics and Traumatology, Spine Disorders Unit, University of Medical Sciences, Poznan, Poland; Department of Cell Biology, University of Medical Sciences, Poznan, Poland; Department of Rehabilitation, Jozef Pilsudski University of Physical Education, Warsaw, Poland; Department of Orthopedics, Pediatric Orthopedics and Traumatology, The Centre of Postgraduate Medical Education in Warsaw, Otwock, Poland

**Keywords:** Estrogen receptors polymorphisms, Age at menarche, Idiopathic scoliosis

## Abstract

**Background:**

The age at menarche (AAM) is commonly in use in patients with IS as one of the maturity indicator suggesting deceleration of the growth velocity. The AAM was suggested to be related to predisposition and curve progression potential of IS. The late age at menarche was reported to be associated with higher prevalence of adolescent idiopathic scoliosis. The age at menarche is determined by both genetic and environmental factors as well as their interactions. Estrogen receptors 1 and 2 polymorphism were reported to be associated with AAM: in *ESR1 XbaI* and *PvuII* site polymorphism and in *ESR2 AluI* site polymorphism.

The purpose of the study was to investigate associations of the *ESR1* and *ESR2* polymorphisms with AAM in IS patients and to evaluate association of AAM with IS severity.

**Methods:**

208 females with IS Caucasian females from Central Europe underwent clinical, radiological and genetic examinations. Four SNPs were selected *XbaI* (A/Grs9340799*)* and *PvuII* (C/T rs2234693*)* in *ESR1* and *AluI* (A/G rs4986938) and *RasI* (A/G rs1256049) in *ESR2*. Samples were analyzed with polymerase chain reaction followed by restriction fragments length polymorphism analysis (PCR-RFLP). The age of a menarche was established during personal interview with the patients and in case of children with their parents. The Cobb angle was measured.

**Results:**

All genotypes followed HWE. Mean AAM for patients was 154.8 ± 14.7 months (12.9 ± 1.2 years). The earliest AAM was 121 and latest 192 months. There was no statistically significant difference between AAM mean values in each genotype, for the *XbaI, PvuII, AluI* and *RsaI* site polymorphisms the p values were p = 0.7141, p = 0.9774, p = 0.7973 and p = 0.2282, respectively. Patients divided according to Cobb into mild (<30°), moderate (30°-49°) or severe (≥50°) IS revealed tendency to delay AAM: 151.9 ± 14.7; 155.2 ± 14.8 and 157.9 ± 14.0 months, respectively. There was statistical significant difference between patients with mild <30° and severe ≥50° IS, p = 0.0267.

**Conclusions:**

In IS patients estrogen receptors polymorphisms did not show association with the AAM. Patients with severe IS form revealed delayed AAM than patients with mild IS form.

**Electronic supplementary material:**

The online version of this article (doi:10.1186/1471-2474-15-383) contains supplementary material, which is available to authorized users.

## Background

Idiopathic scoliosis (IS) is one of the most common spinal diseases in adolescence, affecting 1-3% of adolescent population [1]. This is a three dimensional spine deformity of unknown etiology, consisting of a side curve combined with sagittal plane deviation and axial rotation of vertebrae [1]. The diagnosis is established based on typical clinical signs, confirmed with radiological Cobb angle value of more than 10° on radiological examination while possible reasons for a secondary scoliosis are ruled out [1, 2].

The age at menarche (AAM) is commonly in use in IS patients as one of the maturity indicators suggesting deceleration of the spine growth velocity [3] and decreased risk of curve progression [4]. The AAM was suggested to be related to predisposition to IS and to curve progression potential [5].

A late AAM was reported to be associated with higher prevalence of adolescent idiopathic scoliosis [6]. Nevertheless, the mean age of menarche of IS patients in comparison to healthy population was reported as early, normal, or delayed [5–7].

The AAM is determined by both genetic and environmental factors as well as their interactions [8]. Environmental factors reported are geographic latitude and solar radiation [6], ethnicity [9], nutrition status [10], physical activity [11], living standards [12], father absence [12], stressful events, psychological adjustment [13] and others.

Familial and twin studies suggested that genetic factors have an important influence on AAM [11, 13, 14]. Families observations show that the maternal AAM is associated with daughter**’** s AAM [13]. Towne et al. suggested that approximately half of the phenotypic variation in the timing of menarche among girls from developed countries is due to genetic factors [11].

Research on underlying genetic background of AAM revealed over 25 genetic association studies, 4 linkage analysis and 6 GWAS (genome wide association studies) published [8]. Candidate genes were identified and among them estrogen receptors genes and genes associated with estrogen metabolism were investigated intensively [8]. Estrogens act via estrogen receptors type 1 and type 2 (ESR1 and ESR2) [15].

In the *ESR1* gene two Single Nucleotide Polymorphisms (SNP): rs9340799 (351A > G) and rs2234693 (397C > T) were studied with the *XbaI* and *PvuII* restriction enzymes, respectively. Stavrou et al. in 2002 [16] reported that in Greek females the AAM was significantly delayed in subjects with genotype XX (GG) of the *XbaI* site polymorphism and with genotype XXPP (GGCC, haplotype homozygote) of the *XbaI* and *PvuII* site polymorphisms [16]. Manuck et al. in 2011 [17] described, that coincidence of family environment features and *ESR1* gene polymorphism (GG homozygote for the *XbaI* site polymorphism and CC homozygote for the *PvuII* site polymorphism) may affect age at menarche. Association of the *ESR1 XbaI* and *PvuII* polymorphisms with AAM has not been shown in three publications concerning Caucasian, multi-ethnic and Japanese populations, respectively [18–20].

In the *ESR2* gene two SNPs: rs1256049 (1082G > A) and rs4986938 (1730G > A) were evaluated with the *RsaI* and *AluI* restriction enzymes, respectively. Stavrou et al. [21] reported that females with AA genotype the *AluI* site polymorphism presented the AAM 7 months later than females with AG genotype. They also reported influence of a combination of the *ESR1* and *ESR2* polymorphism on AAM. There are no other studies concerning *ESR2* SNPs and AAM published so far.

*ESR1* and *ESR2* polymorphisms were suggested to present association with predisposition to and severity of IS [22, 23], however two replication studies did not confirm these findings [24–26].

The aim of the study was to investigate association of the *ESR1* and *ESR2* SNPs with AAM in IS patients and to evaluate association of AAM with IS severity.

## Methods

### Material

Two hundred and eight IS Caucasian females from Central Europe (Poland) were recruited. They all underwent clinical, radiological and genetic examinations. Diagnosis of IS was confirmed on standing PA X-rays. Other diseases or reasons for spine curvature were excluded based on history, clinical and radiological examinations. Patients with Cobb angle of 20° and more were included to the study. Fifty seven out of 208 patients (27.4%) underwent surgical treatment due to severe scoliosis. The patients data are described in Table 1.

**Table 1:** **Patients description, N = 208**

Parameter	Mean ± SD	Min - Max
Age at examination [months]	201.5 ± 70.6	148 - 654
Cobb angle [°]	40.2 ± 18.2	20 - 114
Height [cm]	164.2 ± 7.0	149 - 181
Weight [kg]	51.0 ± 9.2	35 - 79
BMI [m/kg^2^]	18.9 ± 2.8	13.7 - 27.5
Age at Menarche [months]	154.8 ± 14.7	121 - 192

### Ethics statement

The study was approved by the Institutional Review Board of the Poznan University of Medical Sciences (No 87/09). The informed consent was obtained from all the patients or their parents in case of children.

### Clinical and radiological evaluation

The AAM was established in personal interview with the patients or their parents in case of children.

The Cobb angle was measured on the PA standing X-ray taken at last follow-up visit or on the X-ray immediately before surgery in case of patients undergoing surgical treatment. The patients were divided into three groups according to Cobb angle value: (1) mild scoliosis (Cobb 20°-29°), (2) moderate scoliosis (Cobb 30°-49°), and (3) severe scoliosis (Cobb ≥50°). The patients were followed up to skeletal maturity unless they underwent surgical treatment. Skeletal maturity was defined as follows: minimum age of 15 years, Risser sign 4 or 5, at least two years after menarche, growth of less than 2 cm during last year. Among the patients there were 80 (38.5%) with Risser sign 5, 91 (43.8%) with Risser sign 4, 10 (4.8%) with Risser sign 3, 5 (2.4%) with Risser sign 2, 9 (4.3%) with Risser sign 1 and 13 with Risser sign 0. Patients with Risser sign 0–3 included to study were surgically treated before the Risser sign 4 was developed. The scoliosis pattern was : single thoracic curve in 67 patients (32.2%), thoracic curve with additional lumbar smaller curve in 78 (37.5%), double thoracic curve in 8 (3.8%), triple curve in 4 (1.9%), single lumbar curve in 14 (6.7%), lumbar curve with additional smaller thoracic curve in 37 (17.7%).

### Genetic analysis

In genetic analysis the four previously reported SNPs were investigated: rs9340799 and rs2234693 for the *ESR1,* and rs4986938 and rs1256049 for the *ESR2.* The genomic DNA was obtained from patients’ peripheral blood samples with AxygenAxy Prep Blood Genomic DNA Miniprep Kit (Axygen Scientific, Inc., Union City, CA, USA). PCR of the selected *ESR1* and *ESR2* gene fragments was performed, using primers described in Table 2.

**Table 2:** **Primers’ description**

Gene	SNP	Enzyme	5’- > 3’	Sequence 5’- > 3’	Annealing temp	Amplicon length
ESR1	rs9340799	*XbaI*	F	CTGCCACCCTATCTGTATCTTTTCCTATTCTCC	71°C	1300 bp
			R	TCTTTCTCTGCCACCCTGGCGTCGATTATCTGA		
	rs2234693	*PuvII*	F	AGGCTGGGCTCAAACTACAG	60°C	759 bp
			R	TCCTTGGCAGATTCCATAGC		
ESR2	rs1256049	*Rsal*	F	TTCTGAGCCGAGGTCGTAGT	66°C	582 bp
			R	TGAATCCTTGGACCCAACTC		
	rs4986938	*AluI*	F	GTGTGTGGTGGGACACAGAG	65°C	646 bp
			R	AGGCCATTGAGTGTGGAAAC		

Restriction Fragments Length Polymorphism (RFLP) analysis for each SNP was carried out. The PCR reaction products were digested with the restriction enzymes (FastDigest Enzyme). Enzymes and restriction sites for each SNP are described in Table 3.

**Table 3:** **RFLP enzymes description**

SNP	Enzyme	Position	Recognition site 5’- > 3’		Allele	Digestion product length
rs9340799	*XbaI*	ESR1	T*CTAGA	T*CT*A* GA	A	910 bp + 390 bp
		Intron 1		TCT*G* GA	G	1300 bp
		351A > G				
rs2234693	*PvuII*	ESR1	CAG*CTG	CAG*C*T* G	T	271 bp + 488 bp
		Intron 1		CAGC*C* G	C	759 bp
		397C > T				
rs4986938	*AluI*	ESR2	AG*CT	*A* G*CT	A	445 pz + 201 pz
		UTR		*G* GCT	G	646 pz
		1730G > A				
rs1256049	*RasI*	ESR2	GT*AC	GT*A C	A	293 pz + 289 pz
		Exon 5		GT*G* C	G	582 pz
		1082G > A				

*Digestion site.

The reaction products were electrophoresed on 2% agarose gel in the presence of ethidium bromide to establish the restriction’s allele. The results were described as AA, AG or GG or for *XbaI*, *AluI and RasI*, and CC, CT or TT or for *PvuII,* depending on presence or absence of digestion, Table 3. Moreover, 30% of randomly chosen samples were reevaluated with the same method (RLFP) and 10% of the randomly chosen samples were sequenced.

### Statistical analysis

For the AAM the mean and SD values were calculated in months. The differences between the mean AAM among the patients grouped according to genotype (for each SNP and for combinations as proposed by Stavrou et al. [16]) and among the patients grouped according to Cobb angle (into Cobb <30°, Cobb 30°-49°, and Cobb ≥50°) were compared with the t-test and one-way analysis of variance (ANOVA). Hardy-Weinberg equilibrium (HWE) was computed with goodness-of-fit Chi^2^ test. A level of the p value <0.05 and CI 95% were considered statistically significant. In the combination of genotypes analysis the Bonferroni correction was applied. The power analysis was performed with G*power v. 3.1 [27].

## Results

**Figure 1:**
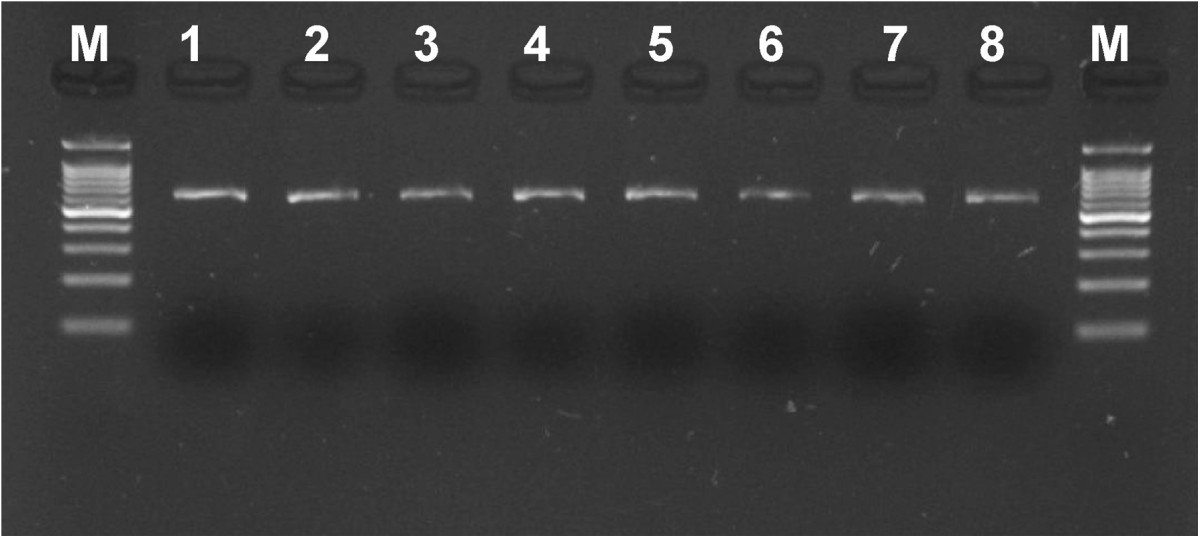
**Example of PCR products electrophoresis for** ***ESR2*** **rs4986938 M – Nova 100 bp DNA ladder size standard (Novazym®), 1–8 – patients DNA.**

**Figure 2:**
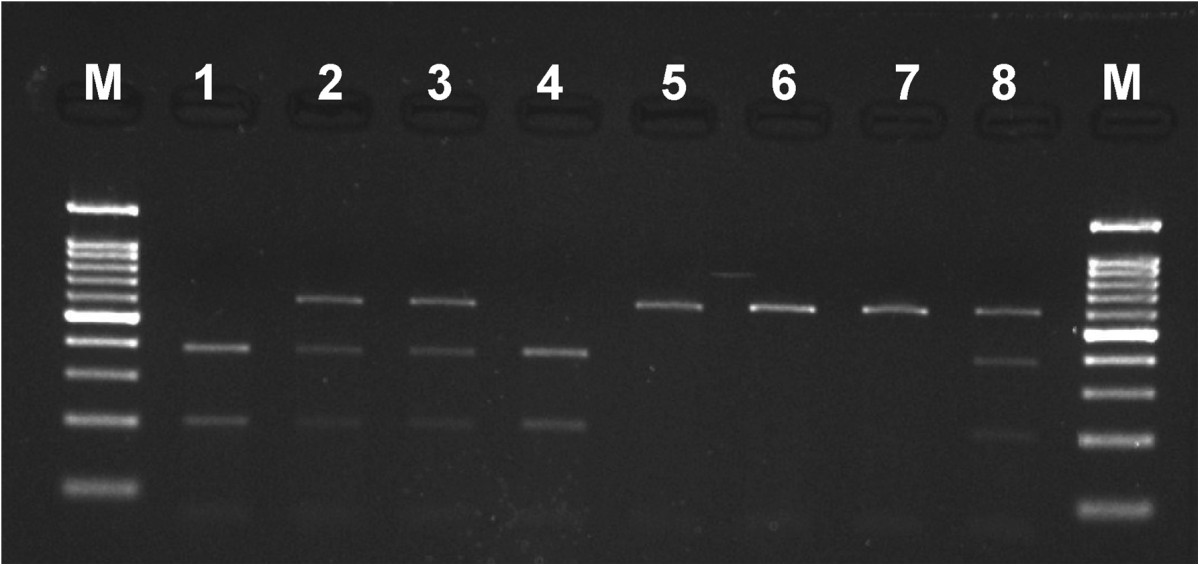
**Example of** ***AluI*** **enzyme RFLP products electrophoresis for** ***ESR2*** **rs4986938 1–8 – patients’ DNA.** M - Nova 100 bp DNA Ladder size standard (Novazym®) (samples 1 and 4 were homozygotes AA genotype – in both alleles enzymatic restriction occurred; sample 5–7 - was homozygote GG genotype – in both alleles enzymatic restriction did not occur; samples 2,3 and 8 – were heterozygotes AG genotype – in one allele enzymatic restriction occurred and in the other did not).

Mean AAM for all IS girls was 154.8 ± 14.7 months (12.9 ± 1.2 years). All genotypes followed HWE (Hardy-Weinberg equilibrium). Examples of PCR products and RFLP products electrophoresis is shown in Figures 1 and 2, respectively. Genotypes distribution, mean and SD values of the AAM are presented in Table 4. No statistically significant difference of the AAM mean values in any studied genotype was found, for the *XbaI, PvuII, AluI* and *RsaI* site polymorphisms the p values were p = 0.7141, p = 0.9774, p = 0.7973 and p = 0.2282, respectively.

**Table 4:** **Genotypes distribution, mean and SD values of the AAM for all patients, N = 208**

SNP	HWE	Genotype	Mean ± SD	Min - Max	P
*XbaI*	0.7429^a^	AA N = 71	153.8 ± 13.4	129 - 191	0.7141^b^
		AG N = 103	155.6 ± 15.9	121 - 192	
		GG N = 34	154.2 ± 13.4	130 - 184	
*PvuII*	0.9733^a^	CC N = 47	154.4 ± 14.2	130 - 192	0.9774^b^
		CT N = 104	154.8 ± 15.7	121 - 192	
		TT N = 57	155.0 ± 13.3	129 - 191	
*AluI*	0.7415^a^	AA N = 26	153.1 ± 14.2	132 - 188	0.7973^b^
		AG N = 92	155.3 ± 14.4	128 - 192	
		GG N = 90	154.8 ± 15.2	121 - 187	
*RsaI*	0.4206^a^	AG N = 22	151.2 ± 13.7	131 - 180	0.2282^c^
		GG N = 186	155.2 ± 14.8	121 - 192	

^a^Chi^2^ test; ^b^ANOVA t; ^c^t-Student test.

In patients divided according to Stavrou et al. [16] into GGCC haplotype homozygotes versus all other haplotypes of the *ESR1* the mean AAM was 153.9 ± 13.3 months versus 154.9 ± 14.9 months, respectively, with a P value 0.7069.

**Figure 3:**
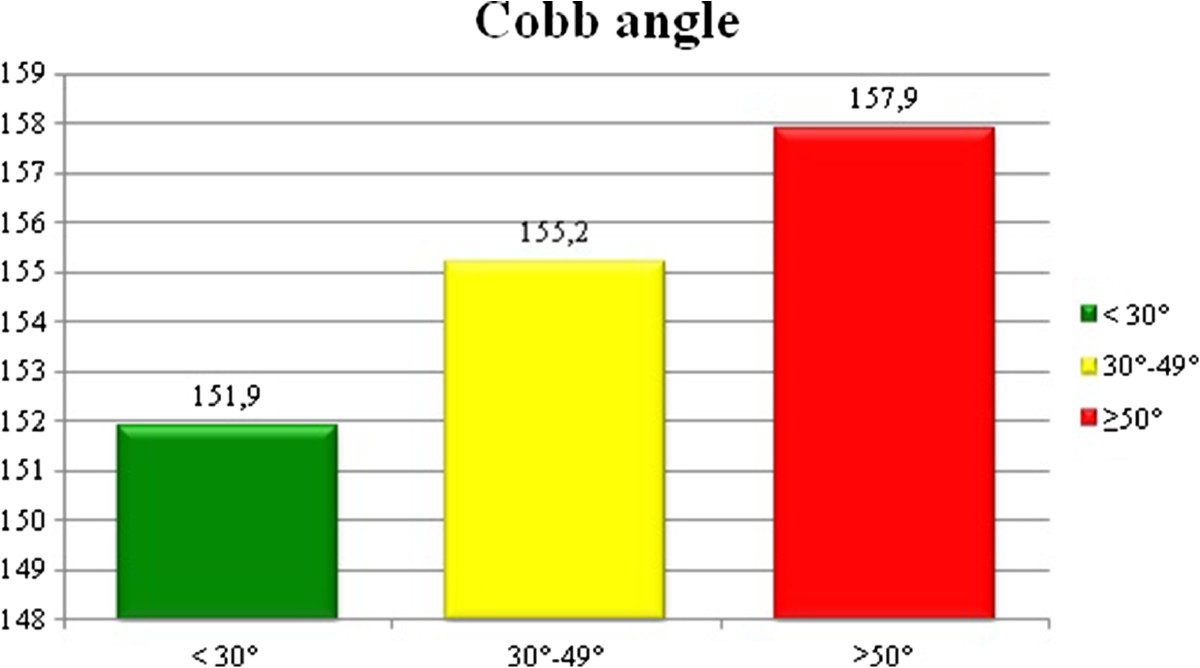
**The mean age at menarche in patients divided according to Cobb angle, p > 0.05.**

In patients grouped according to Cobb angle the mean AAM was: 151.9 ± 14.7 months for patients with mild scoliosis (Cobb <30°), 155.2 ± 14.8 months for patients with moderate scoliosis (Cobb 30°-49°) and 157.9 ± 14.0 months for patients with severe scoliosis (Cobb angle of 50° or more). There was a tendency for delayed AAM with increasing Cobb angle, Figure 3. The AAM difference of 5 months between the mild scoliosis patients and the severe scoliosis patients was statistically significant, Table 5.

**Table 5:** **Mean AAM in patients with mild scoliosis versus patients with severe scoliosis**

Cobb angle	N	Mean ± SD [months]	Min – Max [months]	P
<30°	67	151.9 ± 14.7	121 - 188	0.0267^a^
≥50°	51	157.9 ± 14.0	121 - 191	

^a^t-Student test.

The power analysis revealed that the possible to distinguish difference was 6 months for the *AluI* , *XbaI* and *PvuII* and 9 months for the *RsaI* site polymorphism with the 80% power.

## Discussion

This study evaluated possible association of the age at menarche with selected polymorphisms of two types estrogen receptors genes in a sample of Caucasian females suffering from idiopathic scoliosis. Since the AAM is dependent of both genetic and environmental factors, the potential influence of environmental factors could not be completely eliminated. However, the following parameters were similar throughout the patients of the studied sample: geographical latitude (corresponding to the part of the country presenting less than 5° latitude difference), ethnicity (Polish), nutritional status (BMI ± SD of 18.9 ± 2.8). Although, the IS diagnosis threshold is 10° of Cobb angle [1, 2], in this study only patients with Cobb angle of 20° and more were included to avoid possible bias.

The mean AAM reported for Polish population ranges from 12.65 to 13.13 years [28–30]. Our mean for the whole sample (12.9 years) is placed in the middle of this range. Stavrou et al. found the mean AAM of 12.92 ± 1.26 years [16]. Regardless the latitude and climate differences between Poland and Greece such as intensity of solar radiation, the AAM reported by Stavrou et al. is similar to our result (p = 0.88). The mean BMI in the Stavrou group was 20.7 that is higher of 1.9 comparing to our group, p = 0.0005.

Stavrou et al. reported the AAM to be associated with the *XbaI* site polymorphism. In the adolescent Greek females the study revealed the difference between genotypes XX (GG) and Xx (AG) or xx (AA) which were reported to be of 0.56 years (6.7 months), p = 0.057 and of 0.61 years (7.3 months), p = 0.017, respectively [16]. However, we could not confirm these findings. In this study these differences amounted to 0.12 years (1.4 months) and 0.03 years (0.4 months), respectively, without statistical significance. In the Stavrou et al. study the girls with XP (GC) haplotype homozygotes had the AAM of 0.67 years (8 months) later than all other haplotypes [16]. In this study such a difference was 1 month only, insignificant.

In *ESR2* gene Stavrou et al. reported that girls with the AA genotype in *AluI* site polymorphism had menarche 0.57 years (6.8 months) later than girls with the AG genotype, p = 0.005 [21]. In this study, the biggest difference of the mean AAM between genotypes of *AluI* polymorphism was 0.18 years only (2.2 months) and was not significant. Stavrou et al. have not found any polymorphism in *RasI* restriction site [21]. In this study, the patients with AG versus GG genotype in the *RasI* site polymorphism revealed the biggest noted difference of the AAM of 0.33 year (4 months), not significant.

Allele frequencies may differ among populations and this makes difficult comparing studies from distant countries. Another reason for the results incoherence could be the fact that none of the SNPs investigated by Stavrou et al. was in HWE [16, 21]. Genotypes frequencies in HWE show random distribution in population. Genotypes frequencies do not follow HWE due to nonrandom mating, recent immigration, admixture of different populations, selection or genotyping error [25]. In search for the reasons of differences between the published studies the fact that females with IS versus healthy females were evaluated should be considered. However, in recently published studies concerning the *XbaI* and the *PvuII* site polymorphism no differences in genotypes distribution between the IS patients and healthy control group were shown [24, 25].

A higher progression rate in adolescent females than males suggests that sex hormones may have influence IS curve progression.

Pubertal development and especially pubertal growth spurt is associated with the onset and progression of IS [31]. Many puberty indicators were studied for their association with scoliosis curve progression [2, 5, 31] and among them the AAM is being continuously considered a reliable indicator of a late phase of puberty comprising slowing down pubertal growth [7, 31]. These facts, together with previously reported association of estrogen receptors with IS [22, 23], which was not confirmed in more recent studies [24–26], and with published data concerning the genetic impact on AAM [8, 11, 13, 14, 16, 17, 21], supports searching for a possible cross-talk mechanism. In this study, no association with evaluated SNPs was found, yet linkage between AAM and Cobb angle progression is suggested.

In the study of Grivas et al. the late AAM was parallel to higher prevalence of IS [6]. Those authors suggested that the late onset of menarche correlated with delayed skeletal age and more potential for remaining growth [6]. They argued that prolonged growth period was associated with possibility of scoliosis curve progression [6].

Mao et al. suggested that late menarche may contribute to abnormal pubertal growth and subsequently to modulate curve behavior. Thus, the girls with late onset of menarche may be susceptible to scoliosis progression [5]. In the Chinese population, the IS girls having Cobb angle of more than 60° revealed the onset of menarche at an average age of 13.25 years, which was significantly later than the IS girls with Cobb angle of less than 40° (12.81 years, p < 0.05) [5]. According to Yim et al. in the Chinese population the patients having Cobb angle >40° underwent menarche 5.9 months later than healthy controls, p < 0.05 [32]. In this study, the difference of 6 months of the mean AAM was noted between the IS patients with curves below 30° Cobb angle and the IS patients with curves of 50° and more; the difference was statistically significant, p < 0.05. In all range of results the tendency for a later AAM with increasing Cobb angle was observed. There exist numerous differences between the Asian and the Caucasian populations concerning anthropometric parameters such as height and weight, genotypes distribution [26], as well as geographical latitude and climate parameters. These differences can influence the AAM and may impede direct reference of the data published for the Chinese population [5, 32] to the Caucasian population examined in this study. However, even among populations from the same geographical region incoherence in the AAM can be revealed [6]. In healthy Chinese females the AAM published by Mao et al. and Yim et al. was 12.63 ± 0.98 and 12.14 ± 1.1 years, respectively, which is earlier comparing to the AAM published for Polish females (12.65 - 13.13 years) [28–30]. Regardless the differences in absolute values, the tendency for delayed AAM combined with increased Cobb angle seems to be similar in either population.

The limitation of this study is lack of data potentially influencing the AAM: physical activity [11], living standards [12], father absence [12], and psychological factors [13]. In this study, the healthy control group was not included to compare the AAM; instead the comparison with the recently published data concerning females of the same population was performed.

## Conclusions

In patients with idiopathic scoliosis the estrogen receptors genes polymorphisms did not show association with the age at menarche. The girls with severe IS experienced menarche later comparing to girls with mild IS. Further studies concerning the association of the AAM with progression of IS should address the analysis of additional factors influencing the AAM. This study suggests that attention paid to the AAM may contribute in distinguishing patients with low versus high risk of IS progression.

## Authors’ original submitted files for images

Below are the links to the authors’ original submitted files for images. Authors’ original file for figure 1 Authors’ original file for figure 2 Authors’ original file for figure 3
