# Supplementary figures and images for: Estrogen receptors genes polymorphisms and age at menarche in idiopathic scoliosis
Source: BMC Musculoskelet Disord. 2014 Nov 19;15:383. doi: 10.1186/1471-2474-15-383 (PMC4247216; doi:10.1186/1471-2474-15-383)

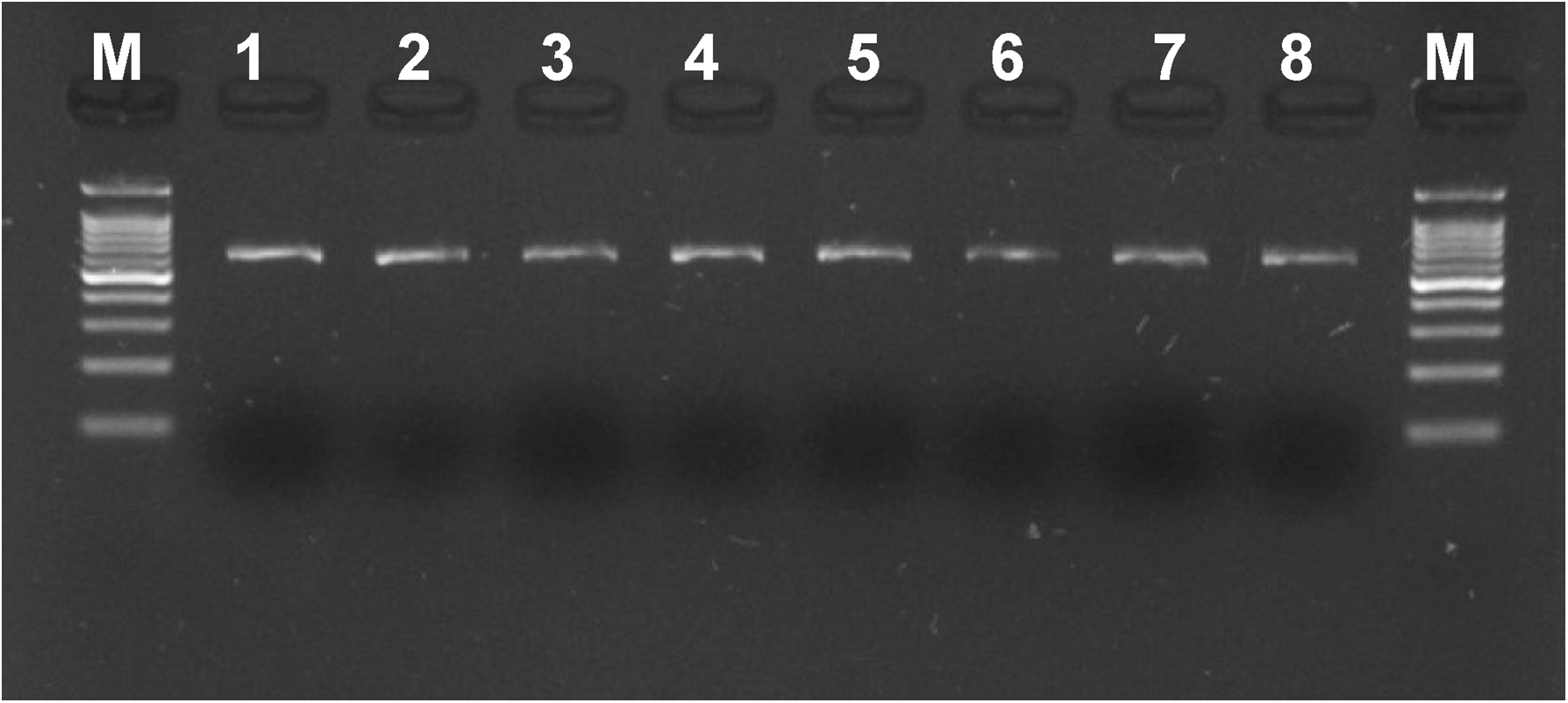

Supplement: Supplementary file 1 — Authors’ original file for figure 1 [file 12891_2014_2309_MOESM1_ESM.tif]

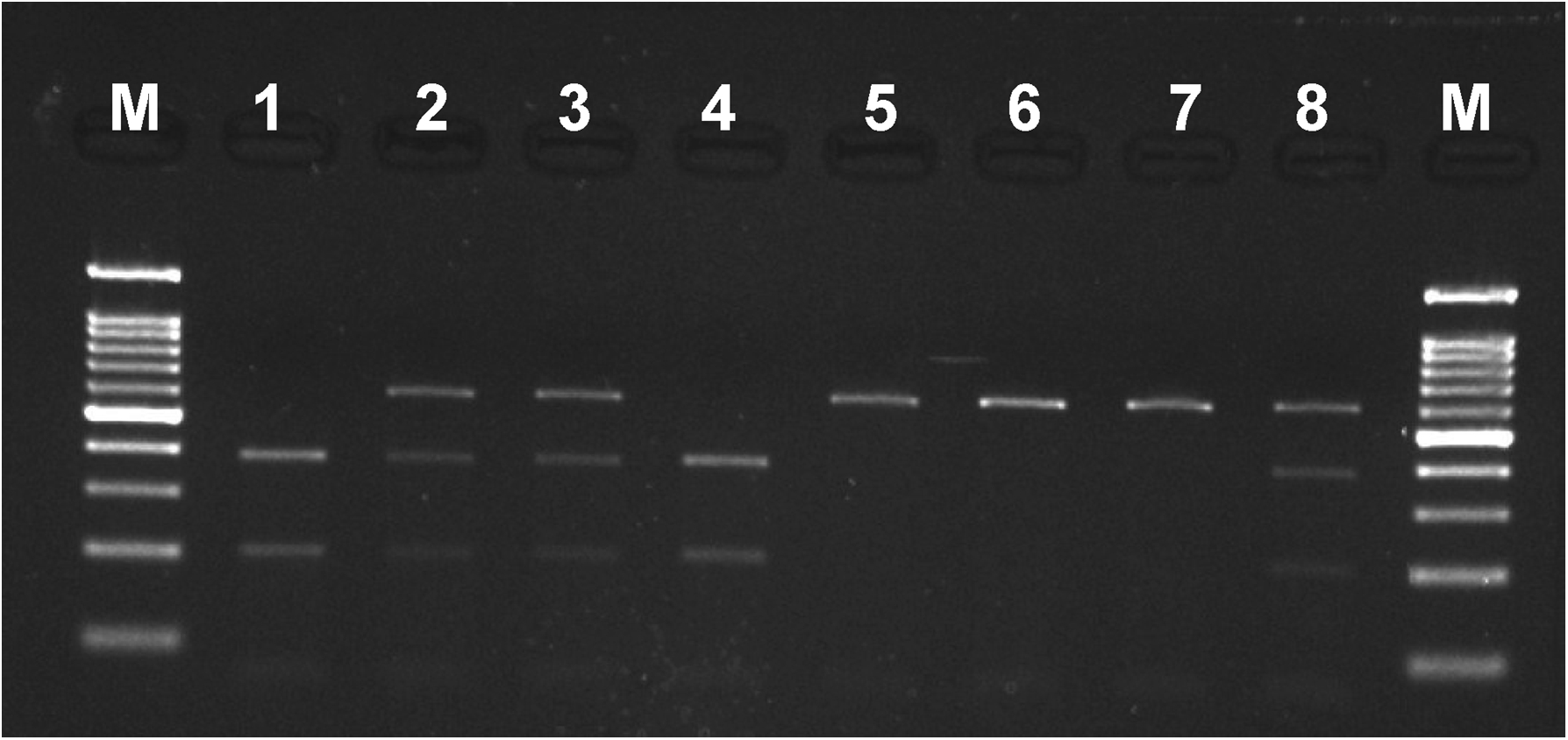

Supplement: Supplementary file 2 — Authors’ original file for figure 2 [file 12891_2014_2309_MOESM2_ESM.tif]

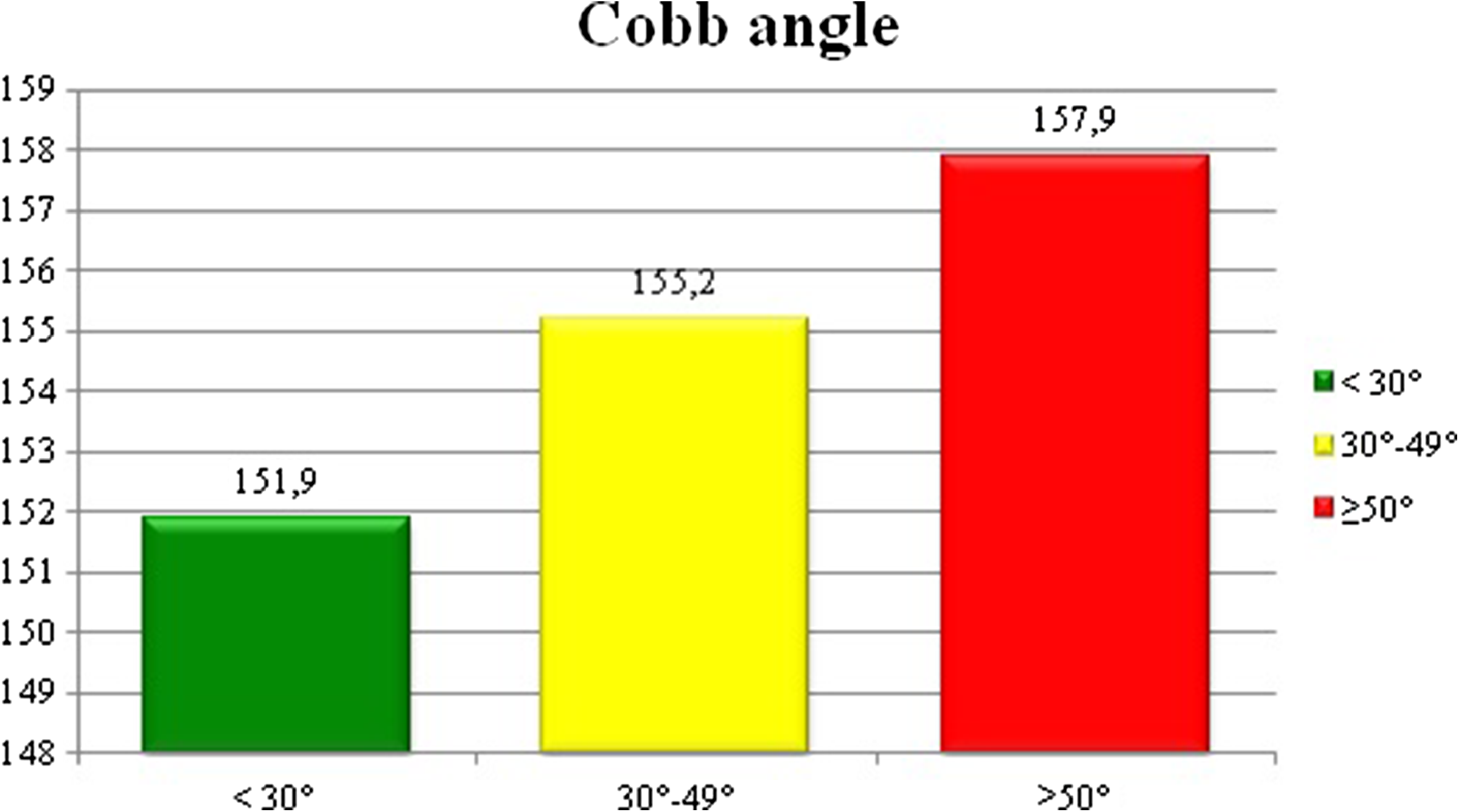

Supplement: Supplementary file 3 — Authors’ original file for figure 3 [file 12891_2014_2309_MOESM3_ESM.tif]
